# Persistent Oscillating Vertigo From Extracranial Venous Compression

**DOI:** 10.1097/ONO.0000000000000065

**Published:** 2025-01-29

**Authors:** Yoon-Hee Cha, Mahmood Gharib, Kayla Chan, Joseph Karam

**Affiliations:** 1 Department of Neurology, University of Minnesota, Minneapolis, Minnesota; 2 Department of Physical Medicine and Rehabilitation, University of Minnesota, Minneapolis, Minnesota; 3 Department of Vascular Surgery, Abbott Northwestern Hospital, Minneapolis, Minnesota.

**Keywords:** Mal de Debarquement Syndrome, MdDS, Venous congestion, Thoracic outlet syndrome

## Abstract

**Background:**

Persistent oscillating vertigo (POV) can be triggered by motion, when it is called mal de débarquement syndrome (MdDS) but can also occur from nonmotion triggers such as neck injury, inflammation, or homeostatic derangements (nonmotion POV [nmPOV]). The pathology underlying MdDS and nmPOV is unknown but shared symptoms include rocking/bobbing/swaying vertigo, headache, cognitive slowing, and fatigue.

**Methods:**

We present a case series of patients with MdDS and nmPOV whose symptoms were relieved after treatment of extracranial venous compression in the neck and thoracic outlet.

**Results:**

POV, regardless of the trigger, was associated with compressions at one or more of the following locations: 1) internal jugular vein (IJV) between the transverse process of the atlas and the styloid process, 2) IJV under the sternocleidomastoid muscle (SCM), and 3) subclavian vein at the thoracic outlet. Compressions were typically bilateral and in tandem. Catheter venography showed dynamic obstruction and shunting of venous blood to the petrosal sinuses around the inner ear and the vertebral veins. Relief of these obstructions with styloidectomy, stenting, neurotoxin to SCM and anterior scalene, SCM partial myotomy, and thoracic outlet decompression significantly relieved POV even with unilateral decompression.

**Conclusions:**

Oscillatory perceptions of POV whether MdDS or nmPOV may be from the detection of low amplitude pulsations induced by venous outflow obstruction in areas of high freedom of motion (occipito-atlanto junction, mid-neck, and thoracic outlet). Impaired venous outflow and raised venous pressure around the inner ear could lead to continuous peripheral stimulation of the vestibular system and downstream central effects.

Mal de débarquement syndrome (MdDS) has been hypothesized to be a disorder of entrainment to passive motion, typically experienced during water or air travel (1,2). The slow oscillating frequency of wave motion is theoretically conducive to entrainment but why motion exposures as short as a few hours can lead to years of being trapped in that entrained state has no mechanistic explanation (3,4). A similar clinical syndrome of persistent oscillating vertigo (POV) occurs without a motion trigger (nonmotion POV [nmPOV]) typically after a neck injury, inflammation, and homeostatic derangements. nmPOV has a higher association with migraine and is more resistant to treatment than MdDS (5,6). However, the symptoms of MdDS and nmPOV overlap, including rocking/bobbing/swaying vertigo, headache, cognitive slowing, and fatigue. Both lead to profound disability (7–10). Despite the description of wavelike motion and the strong comorbidity with headache, venous outflow obstruction has so far not been considered an underlying mechanism for POV. This pathology, however, may explain many of the clinical features of POV and be a potential new target for treatment.

## OBJECTIVE

We present 6 cases of POV that resulted from extracranial venous compression and were relieved by targeting this pathology. Persistent MdDS was diagnosed according to Bárány Society criteria (1) and nmPOV when symptoms were similar without a motion trigger. Brief clinical histories are presented in the following sections. Only the relevant positive exam findings are presented; complete neurological exams were otherwise normal. Upper limb tension tests (numbness/tingling of hand induced when arms are abducted and the head is tilted contralaterally), anterior scalene tenderness, and excluding other arm pathology were used to diagnose thoracic outlet syndrome (TOS) (11). Venous TOS was diagnosed based on exam findings suggesting venous congestion and verified with imaging (ultrasound [US], computed tomography, venography). The authors used quantitative Doppler US with dynamic arm movements and a computed tomography venogram of the head and neck including the upper chest to diagnose vein compressions. All patients underwent a course of physical therapy before escalating to injections with neurotoxin or surgical interventions.

## METHODS

Patients were identified through the institutional review board study EVENHEAD (Extracranial Venous Narrowing in Headache and Dizziness) of which the lead author is the principal investigator. A selection of patients from that study is presented based on the combination of imaging data, adequate follow-up, and patient permission.

## PATIENTS

### Patient 1: MdDS (Bilateral IJV Compression at C1 + Neurogenic TOS + Left Renal Vein Compression)

A 40-year-old woman presented 9 months after she developed POV after a yacht ride. The POV was rated as 10/10 and was associated with severe head pressure, ear pressure, neck stiffness, and cognitive slowing. It resolved when riding in a car. Prolonged computer use, visual stimulation, barometric pressure changes, and menses worsened symptoms. The patient was also diagnosed with pelvic congestion syndrome and left renal vein compression. Exam: subjective rocking frequency of 0.8 Hz with a heart rate of 80 bpm. The neck was anteroflexed. She had bilateral positive upper limb tension tests, with the symptomatic hand getting numb and cold.

### Patient 2: MdDS (Unilateral IJV Structural Lesion + Venous TOS)

A 36-year-old woman presented 8 months after developing pulse synchronous POV after sleeping overnight on a yacht. The POV was rated as 3–5/10 and decreased with driving but worsened with lying down. She suffered from left-side predominant migraine headaches that were focused around the left eye, left skull base, and left neck and arm. There was left eye blurred vision. Exam: swelling of the left hand, fatiguing weakness of the left arm and hand with abduction, and positive upper limb tension test bilaterally, worse on the left side.

### Patient 3: MdDS (Bilateral Venous TOS)

A 42-year-old man presented 15 months after developing POV following several days of water-based activities such as snorkeling and boating. The POV severity level was 2–8/10 depending on daily stressors. Symptoms improved with riding in a car. He noted head pressure when bending over and raising his arms. Exam: bilateral positive upper limb tension tests.

### Patient 4: nmPOV (Bilateral IJV at C1 + TOS)

A 40-year-old man developed POV in his teen years, which worsened significantly after a respiratory infection 2 years before presentation. There was no motion trigger. The POV worsened with arm abduction, particularly the right arm. Exam: positive bilateral upper limb tension tests.

### Patient 5: nmPOV (Bilateral IJV Entrapment by SCM + TOS)

A 31-year-old woman with a history of migraine headaches presented 5 months after the onset of POV that occurred in the setting of rapidly worsening migraine headaches. Symptoms were worse with any head movement and when riding in a car. The migraines were experienced as pressure over the eyes, cheeks, and ears and were worse on the right side. There were no aural symptoms. Exam: positive upper limb tension tests, worse on the right side.

### Patient 6: nmPOV (Bilateral IJV Entrapment by SCM + TOS)

A 55-year-old woman presented 9 months after developing POV following a respiratory infection. Symptoms were better when riding in a car. This worsened after a limb surgery and then again after a ferry ride. There was severe cognitive slowing, tremulousness, speech difficulty, and faintness. Symptoms worsened with head extension and head turning, particularly to the left. There was no history of migraine but a long-standing history of neck and shoulder tension and arm paresthesia when they were outstretched. She had bilateral predominantly left-sided nonpulsatile tinnitus, worse when reclined. Exam: leftward laterocollis and torticollis and a positive upper limb tension test on the left side.

**FIG. 1.**
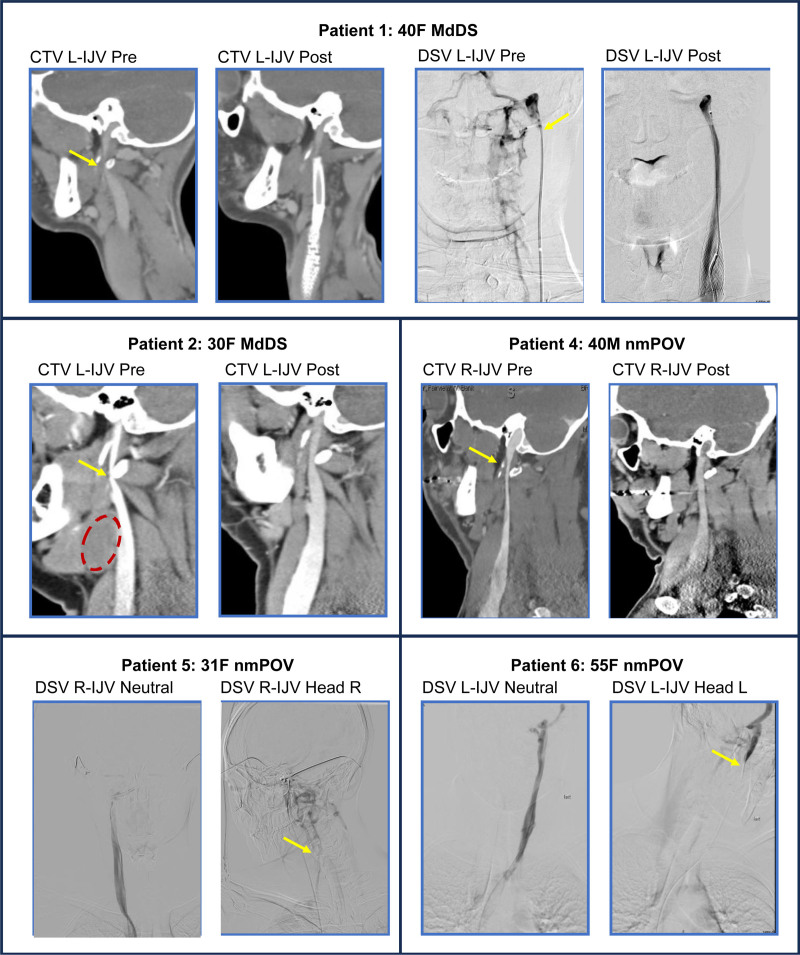
Imaging studies baseline and after treatment in select cases. Dotted oval in patient 2 encircles the branchial cyst, and yellow arrows indicate points of compression/obstruction. Patients 1, 2, and 4 had IJV compressions at C1. Patients 5 and 6 had muscular compressions of the IJV induced by head turning. All patients had concurrent TOS. CTV indicates computed tomography venogram; DSV, digital subtraction venogram; IJV, internal jugular vein; L, left; R, right; TOS, thoracic outlet syndrome.

Relevant imaging reports, interventions, results, and duration of follow-up are presented in Table 1. Imaging pertinent to each case is presented in Figure 1.

**TABLE 1.** Diagnostic evaluations, treatment, and results

	Quantitative Doppler ultrasound	CTV head and neck	Catheter venography	Treatment	Result	Follow-up since last procedure
Patient 1: MdDS 40 years, female	Borderline right SCV velocity in the neutral position and >80% decreased velocity of the right IJV with the head turned to the left	Mild right and moderate left IJV compression of the IJV at atlas (C1) under the styloid process, bilateral occlusion with head flexion. Right styloid 56 mm, left 41 mm	Critical stenosis left IJV at C1–3, dilated suboccipital venous plexus with collaterals, 8 mm Hg right IJV gradient with head turned right, 5 mm Hg left IJV gradient head turned left	Left IJV stent then left styloidectomy + posterior belly digastric resection	80%↓ POV, 50%↓ cognitive slowing. Accessory nerve injury from stent resolved after 6 months	1 year
Patient 2: MdDS 30 years, female	Bilateral reduction of SCV velocity at 90 degrees, low velocity of the left IJV in head flexion, extension, and head left	Enlarged branchial cyst abutting the left IJV	None	Branchial cyst resection then left first rib resection + scalenectomy	100%↓: 70%↓ POV w/branchial cyst resection. 30%↓ POV + left-sided migraines 100%↓ w/left first rib resection + scalenectomy	2.5 years
Patient 3: MdDS 42 years, male	Severely reduced right SCV and completely occluded left SCV flow on arm abduction	Normal	None	Neurotoxin to bilateral SCM + ASM, then left first rib resection + scalenectomy	>80%↓ POV with both neurotoxin and surgery. Improvement more sustained with surgery	8 months
Patient 4: nmPOV 40 years, male	Complete obstruction of bilateral SCV at 90 degree abduction, no flow in the left IJV in head left and head extension	Severe bilateral compression of the IJV at C1	Left IJV occluded at C1. Right IJV occluded with head turn bidirectionally at C1, contrast shunting to the vertebral veins, brainstem, and spinal cord	Right side styloidectomy + posterior belly digastric resection	70%↓ POV. Residual POV triggered by right arm abduction	2 years
Patient 5: nmPOV 31 years, female	Right SCV waveform flattening 90 and 135 degrees. Right IJV occluded in head right position. Left IJV occluded in extension	Bilateral IJV compression by the SCM in neck extension	Right IJV occluded head right position, collateralization, and induction of a 7-mm Hg pressure gradient	Right SCM partial myotomy, first rib resection, scalenectomy, and pectoralis minor tenotomy	80%↓ POV. Residual POV triggered by repeated head turning. Migraines improved daily to perimenstrual	2 years
Patient 6: nmPOV 55 years, female	Small drop in velocity in both SCVs at 90 degrees, otherwise normal	Mild to moderate narrowing of bilateral IJVs at C1 and under the SCM worsened with head extension	Retrograde flow of left IJV into the transverse sinus in neutral, worsened on head left. Right IJV flow normal in neutral, obstructed and shunted to vertebral veins with head right	Neurotoxin to bilateral SCM + ASM	80%↓POV peak Botox effect. Scheduled for SCM partial myotomy	9 months

↓ indicates reduction in symptoms.

ASM indicates anterior scalene muscle; IJV, internal jugular vein; MdDs, mal de débarquement syndrome; nmPOV, nonmotion POV; POV, persistent oscillating vertigo; SCM, sternocleidomastoid muscle; SCV, subclavian vein.

## CONCLUSIONS

We present an illustrative case series of a spectrum of patients with persistent MdDS and nmPOV successfully treated by targeting extracranial venous compressions. Venous compressions were of the internal jugular vein (IJV) at the atlas and/or under the sternocleidomastoid (SCM) muscle with concurrent compression of the subclavian vein (SCV) at the thoracic outlet. Compressions were nearly always bilateral and in tandem, though typically worse on one side. This suggested that the symptomatic state occurs from cumulative loss of collateral venous outflow that surpasses a compensation threshold. A lower burden of these compressions may be present at baseline, which is well-compensated and asymptomatic until there is a motion or nonmotion challenge. The neck strain induced by oscillating motion, such as on a boat, may lead to compression at both the skull base and under the SCM because of compensatory neck movements made for the oscillating boat motion. This may be due to the reverse pendulum effect when the feet are planted on a moving platform and the neck must make continuous adjustments to maintain a stable head position. These compressions occur in areas of high freedom of motion and can also be induced by postural strain (eg, cramped airplane seat), neck injury (eg, concussions/whiplash), or inflammation (eg, infections). Concurrent TOS that lead to SCV compression may reduce the capacity for alternative collateral venous drainage through the external jugular vein, which drains into the SCV. Thus, relieving even one side of the compression can relieve more than half of the symptom burden. In our patients, symptoms could be retriggered with postural strain of the neck or with arm movements, consistent with the location of the compressions at areas of freedom of motion.

Extracranial venous compression could raise intracranial and intra-aural pressure leading to downstream effects of continuous vestibular irritation as noted in the venograms showing retrograde venous flow to the petrosal sinuses and vertebral veins induced by head turning. This model better aligns with the pathophysiology of pulsatile tinnitus rather than a model of motion maladaptation (12). Unlike most causes of pulsatile tinnitus, however, the major compressions in POV were extracranial. This may explain why POV can be suppressed with passive motion, which may have an effect similar to white noise in tinnitus but is worsened with head motion that exacerbates the compressions.

The frequency of perceived oscillation in MdDS/nmPOV could be but was not usually pulse synchronous. This may be due to the low pulse pressure of the venous system and the modulation of venous pressure by respiration and body position that lead to more beat-by-beat variability than in arterial pulsations (13–15). Unlike arterial pressure, venous pressure is very much affected by hydrostatic pressure (column of water effects) that changes with postural changes (16). POV can worsen when the patient is reclined, which is atypical for most balance disorders but occurs in almost 30% of patients with MdDS/nmPOV (7). This paradoxical phenomenon could be secondary to raised venous pressure in the reclined position when venous drainage of the head is not augmented by gravity. Compression of the IJV, which is the preferred venous outflow path in the reclined position (versus the paravertebral venous plexus in the upright position), maybe a clue to IJV flow compromise when POV is worsened in recumbency (17).

This vascular model better explains the greater prevalence of POV in females who are more prone to venous disease (transverse sinus stenosis, hypercoagulability, pelvic venous congestion, etc) than males as well as the typical mid-life age of onset of MdDS/nmPOV, which represents cumulative years of musculoskeletal wear and tear. The process of venous congestion can have both local effects on the inner ear, as well as generalized effects on cerebral fluid dynamics can thus explain the broad spectrum of symptoms in POV such as headaches, neck stiffness, fatigue, and cognitive problems (4,6). The properties of the venous system that make it function as an oscillation dampener of physiologic challenges (hydration, barometric pressure, weight, position, etc) may help explain the many kinds of intrinsic and extrinsic perturbations that make MdDS/nmPOV worse. Symptom exacerbations may represent the capacitance system reaching its limits of compensation during homeostatic strain.

The extracranial venous compression model for POV may open more avenues for treatment based on an observable and measurable structural model rather than a theoretical one of maladaptation. These include neck physical therapy, neurotoxin injections, and surgery in select cases. The historical lack of focus on this structural pathology may explain the extreme resistance to the treatment of both MdDS and nmPOV. Moreover, this model may explain previous observations of central functional connectivity alterations, vestibular-ocular reflex cross-coupling, and prolonged vestibulo-ocular reflex time constants in POV, which may all be downstream effects of venous pressure changes around the inner ear and brainstem. Thus, many cases of MdDS/nmPOV may not be from entrainment to a vestibular stimulus but rather from continuous activation of the vestibular system (18,19).

We present these limited cases as proof of principle rather than illustrative of the prevalence of venous pathology in POV. As such, the age and sex distribution of the patients presented do not cleanly align with prior reports in which there is a female predominance with a peak onset in the 5th decade (7,20). These cases are meant to generate hypotheses to explain an intractable syndrome with unexplained features. The venous abnormalities presented are also not exhaustive of the kinds of abnormalities that can be present in extracranial venous compression syndromes. Prevalence can only be determined if venous pathology is systematically and broadly evaluated in patients who present with POV (head, neck, chest, abdomen). These evaluations should be considered in treatment-refractory cases since addressing significant underlying venous compressions may help make some intractable symptoms more manageable.

## FUNDING SOURCES

None declared.

## CONFLICT OF INTEREST STATEMENT

None declared.

## DATA AVAILABILITY STATEMENT

Data are not available for review due to patient confidentiality. These data can not be anonymized when shared.
